# TYRP1 mRNA level is stable and MITF-M-independent in drug-naïve, vemurafenib- and trametinib-resistant BRAF^V600E^ melanoma cells

**DOI:** 10.1007/s00403-019-01995-w

**Published:** 2019-10-17

**Authors:** Mariusz L. Hartman, Malgorzata Czyz

**Affiliations:** Department of Molecular Biology of Cancer, Medical University of Lodz, 6/8 Mazowiecka Street, 92-215 Lodz, Poland

**Keywords:** TYRP1, MITF, miR sponge, Melanoma, Resistance, Targeted therapy

## Abstract

TYRP1 mRNA is of interest due to its potential non-coding role as a sponge sequestering tumor-suppressive miRs in melanoma. To our knowledge, there is no report on changes in *TYRP1* expression in melanomas after development of resistance to targeted therapies. We used patient-derived drug-naïve RAS^Q61R^ and BRAF^V600E^ melanoma cell lines. In BRAF^V600E^ melanoma cells, resistance to vemurafenib and trametinib was developed. A time-lapse fluorescence microscope was used to rate proliferation, qRT-PCR and Western blotting were used to assess *TYRP1* expression and MITF-M level and activity. A high TYRP1 protein level in RAS^Q61R^ cells corresponded with high TYRP1 mRNA level, whereas undetectable TYRP1 protein in BRAF^V600E^ cells was accompanied by medium mRNA level, also in cells carrying NF1^R135W^ variant in addition. *TYRP1* expression was MITF-M-independent, since similar transcript status was found in MITF-M^high^ and MITF-M^low^ cells. For the first time, we showed that *TYRP1* expression remained unaltered in melanoma cells that became resistant to vemurafenib or trametinib, including those cells losing MITF-M. Also drug discontinuation in resistant cells did not substantially affect *TYRP1* expression. To verify in vitro results, publicly available microarray data were analyzed. TYRP1 transcript levels stay unaltered in the majority of paired melanoma samples from patients before treatment and after relapse caused by resistance to targeted therapies. As TYRP1 mRNA level remains unaltered in melanoma cells during development of resistance to vemurafenib or trametinib, therapies developed to terminate a sponge activity of TYRP1 transcript may be extended to patients that relapse with resistant disease.

## Introduction

Melanoma is the most deadly form of skin cancer. One of the important factors determining melanoma risk is the epidermal melanin content [45]. TYRP1 (tyrosinase-related protein 1) and two other enzymes, tyrosinase and TYRP2, are active in the melanin production. TYRP1 is a marker of melanocyte differentiation but it is also involved in the survival response to oxidative stress [16]. TYRP1 protein expression is reduced in invasive melanomas [4], while TYRP1 mRNA level is increased [28]. TYRP1 mRNA, in addition to the protein-coding function, has been recognized as contributing to regulation of gene expression by serving as an endogenous miR sponge [17].

In melanocytes, expression of about one hundred genes, including *TYRP1*, is microphthalmia-associated transcription factor (MITF)-dependent [6, 14, 18, 33, 42]. The M isoform of MITF (MITF-M) is one of the major players affecting melanoma phenotype [5, 20, 26]. Its regulation is complex and involves microenvironmental components [20, 24]. A high MITF-M level is connected with differentiation, a medium level with proliferation, whereas a low level is associated with an invasive and stem-like phenotype [5, 20, 26]. MITF-M also plays a prosurvival role in melanoma cells [21]. The MITF level is enhanced in BRAF^V600E^ melanoma cells upon acute exposure to vemurafenib [35], and MITF inhibition increases sensitivity of cells to this inhibitor [1]. On the contrary, the MITF level and expression of several MITF-dependent genes are markedly reduced in vemurafenib-resistant melanomas resulting in more primitive phenotypes of melanoma cells [40]. Reduced *MITF* expression followed by the suppression of MITF-dependent pigmentation program were recently reported not only in vemurafenib-resistant cell lines but also in most of trametinib-resistant cell lines [8].

Therefore, we found it interesting to investigate changes of TYRP1 transcript levels in relation to MITF level and its activity shown as transcript levels of other MITF-dependent genes, *SLC45A2*, *BIRC7*/livin and *BCL2A1*, during the development of drug resistance. *SLC45A2* (solute carrier family 45), together with *TYRP1* belongs to pigmentation-related genes [44], whereas *BIRC7* (baculoviral IAP repeat‐containing 7) and *BCL2A1* (BCL2-related protein A1) encode prosurvival proteins [9, 32]. We assumed that diminution of MITF-M level during development of resistance would be accompanied with reduced expression of MITF-M-dependent genes. The question was whether TYRP1 mRNA would also be reduced. The answer is important as reduced level of the TYRP1 transcript may limit its function as a miR sponge in resistant cells. We performed our study in drug-naïve MITF-M^high^ and MITF-M^low^ patient-derived melanoma cell lines and their vemurafenib- or trametinib-resistant counterparts, also subjected to drug discontinuation (‘drug holiday’).

## Materials and methods

### Drugs

Vemurafenib and trametinib were purchased from Selleck Chemicals LLC (Houston, TX, USA).

### Melanoma cell line generation and culture

Tumor tissues from drug-naïve melanoma patients were processed as described previously [22]. The study was approved by Ethical Commission of Medical University of Lodz and informed consent was obtained from all individual participants included in the study. Melanoma cells were maintained in culture as described previously [37]. To generate lines resistant to vemurafenib or trametinib, cells were cultured for 4–5 months with increasing concentrations of drugs, from 1 to 10 µM and from 1 to 50 nM, respectively. For ‘drug holiday’ experiments, the drug was removed from the medium for 10 days.

### A time-lapse fluorescence microscopy

Melanoma cells were grown in 96-well plates at 8 × 10^3^ cells/well. For cell proliferation, a time-lapse fluorescence microscope system (IncuCyte, Essen Bioscience) was used. The data were analyzed using the IncuCyte Zoom original software. Proliferation was assessed as changes in the area occupied by cells (% of confluence) over time. It was expressed as % of confluence of cells at indicated time divided by % of confluence of cells at time 0.

### RNA isolation and quantitative real-time PCR (qRT-PCR)

Extraction of RNA, cDNA synthesis and qRT-PCR were described previously [22]. Primer sequences are shown in Table 1. To calculate the relative normalized expression of target genes, a reference gene *RPS17* and a mathematical model including an efficiency correction were applied.

**Table 1:** Primer sequences, forward (F) and reverse (R) used in the qRT-PCR experiments

Gene	Sequence	*T*_M_ (^o^C)	Amplicon (bp)
*BCL2A1*	F: GGATAAGGCAAAACGGAGGCTG	62	183
	R: CAGTATTGCTTCAGGAGAGATAGC	59	
*BIRC7*	F: TGTCCACAGTGTGCAGGAGACT	64	127
	R: GGCACTTTCAGACTGGACCTCT	64	
*MITF-M*	F: GCTGGAAATGCTAGAATA	57	379
	R: TTCCAGGCTGATGATGTC	59	
*RPS17*	F: AATCTCCTGATCCAAGGCTG	60	142
	R: CAAGATAGCAGGTTATGTCACG	58	
*SLC45A2*	F: CTTTGCATCAGCCACCTCATTGG	65	153
	R: TCCAACCTCGACTCCTCTTTCG	64	
*TYRP1*	F: GAAAAGAGCCACTTTGTCAGGG	62	104
	R: CCATCTGGTCCCAGTATGTCT	61	

### Cell lysate preparation and Western blotting

Cell lysate preparation and Western blotting were described elsewhere [34]. Antibodies detecting MITF (Cell Signaling, Danvers, MA, USA), TYRP1, GAPDH (Santa Cruz Biotechnology, Santa Cruz, CA, USA), or β-actin (Sigma-Aldrich) were used followed by binding of HRP-conjugated anti-mouse/anti-rabbit antibodies (Santa Cruz Biotechnology). The proteins were visualized using ChemiDoc Imaging System (Bio-Rad).

### Analysis of *TYRP1* expression reported in data sets from the Gene Expression Omnibus (GEO) database

The publicly available microarray data sets (accession numbers: GSE77940, GSE61992, GSE50509 and GSE99898) were downloaded from the GEO database (https://www.ncb.nlm.nih.gov). The *TYRP1* expression profiles were developed from paired BRAF^V600^ melanoma samples from 31 patients in pretreatment stage and after relapse due to development of resistance to either vemurafenib or dabrafenib, and from paired melanoma samples from 17 patients before treatment and after relapse due to resistance to a combination of dabrafenib and trametinib. Gene expression values were log_2_ transformed.

### Statistical analysis

Graphs represent mean ± SD of three biological replicates. Student’s *t* test was used to determine significant differences between the mean values. The difference was considered significant if *p* ≤ 0.05.

## Results

### TYRP1 expression in patient-derived melanoma cell lines is MITF-M-independent

**Fig. 1:**
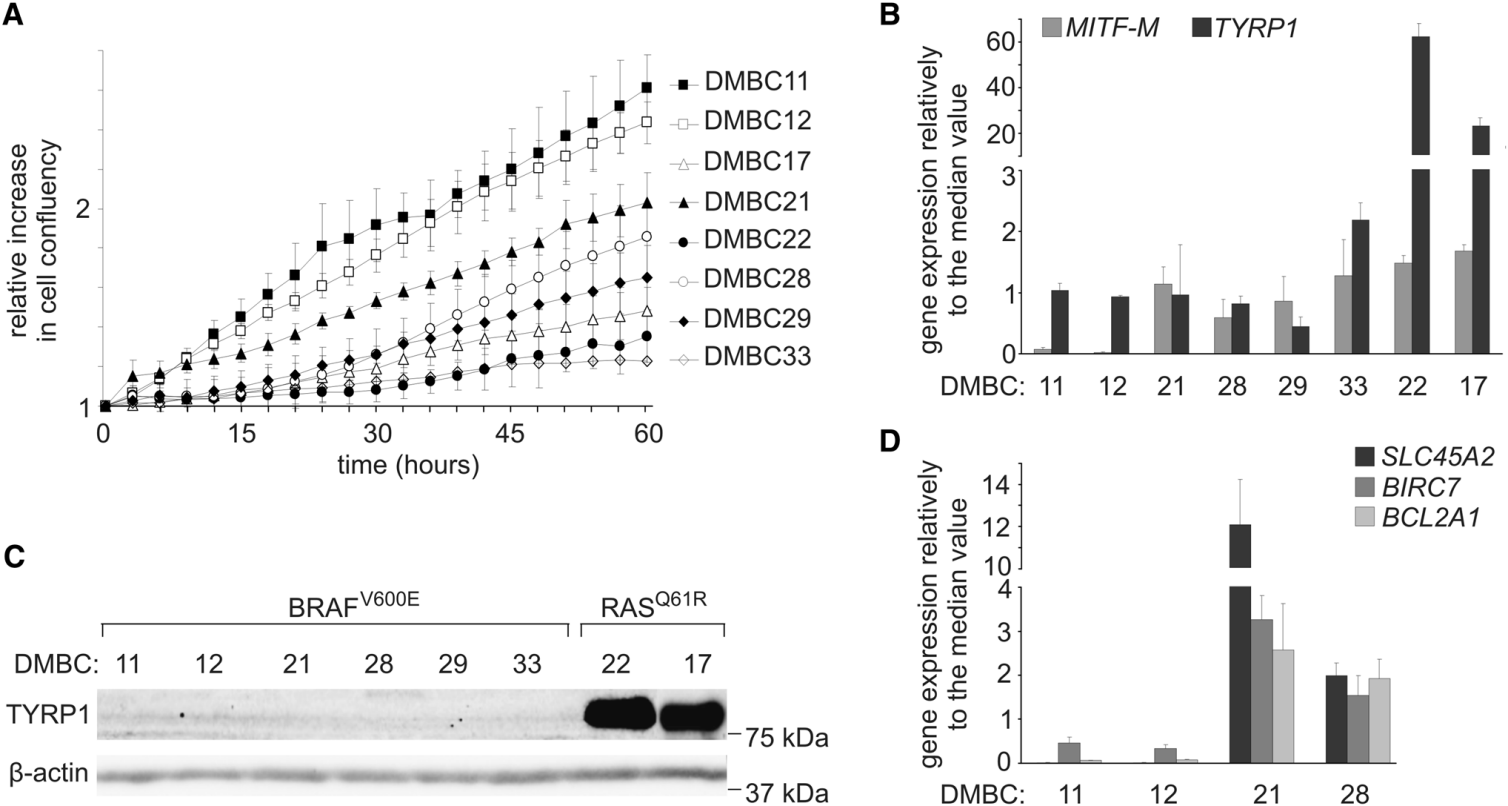
TYRP1 transcript level in patient-derived melanoma cell lines is MITF-M-independent and not related to proliferation rate. **a** Proliferation time-courses. Cell proliferation was monitored by analyzing the occupied area of cell images over time using IncuCyte, and it is shown as an increase in cell confluency relative to the confluency at time 0. *n* = 3 **b** Expression of *TYRP1* and *MITF-M* was determined by qRT-PCR and normalized to the expression of a reference gene *RPS17*. Gene expression levels in each melanoma cell line are expressed relative to the median value of all eight cell lines. Bars represent mean values ± SD, *n* = 3. **c** Representative Western blot images showing basal levels of TYRP1 protein in tested melanoma cell lines. β-Actin was used as a loading control. **d** Expression of three MITF-dependent genes, *SLC45A2*, *BIRC7* and *BCL2A1*, was determined in two MITF-M^low^ and two MITF-M^high^ melanoma cell lines by qRT-PCR and normalized to the expression of a reference gene *RPS17*. Gene expression levels in each melanoma cell line are expressed relative to the median value of all four cell lines. Bars represent mean values ± SD, *n* = 3

Eight patient-derived melanoma cell lines were used initially in this study. Six of them harbor a mutation leading to BRAF^V600E^, and two of them harbor a RAS variant, either NRAS^Q61R^ (DMBC22 cell line) or HRAS^Q61R^ (DMBC17 cell line) [23]. DMBC28 cell line harbors an NF1^R135W^ variant in addition to BRAF^V600E^ alteration [23]. They exerted different proliferation rates as assessed by time-lapse fluorescence microscopy (Fig. 1a). *TYRP1* expression was MITF-M-independent, since in all BRAF^V600E^ melanoma cell lines, both MITF-M^high^ and MITF-M^low^, levels of TYRP1 transcript were similar (Fig. 1b). Interestingly, TYRP1 mRNA levels in cells carrying mutation either in *NRAS* or in *HRAS* were much higher than in BRAF^V600E^ melanoma cells. This was well reflected at the protein level as TYRP1 protein was undetectable in BRAF^V600E^ melanoma cells, whereas for RAS^Q61R^ cells a strong signal was obtained (Fig. 1c).

To verify whether only *TYRP1* expression was MITF-M-independent, the transcript levels of other genes, *BIRC7, BCL2A1* and *SLC45A2,* previously recognized as regulated by MITF-M, were assessed in MITF-M^low^ and MITF-M^high^ cell lines. Transcript levels of these genes in MITF-M^low^ cell lines were substantially lower than in MITF-M^high^ cell lines (Fig. 1d). These results indicate that among the genes whose expression was evaluated only *TYRP1* was MITF-M-independent.

### TYRP1 transcript level is similar in drug-naïve, vemurafenib- and trametinib-resistant melanoma cells, also those on ‘drug holiday’

**Fig. 2:**
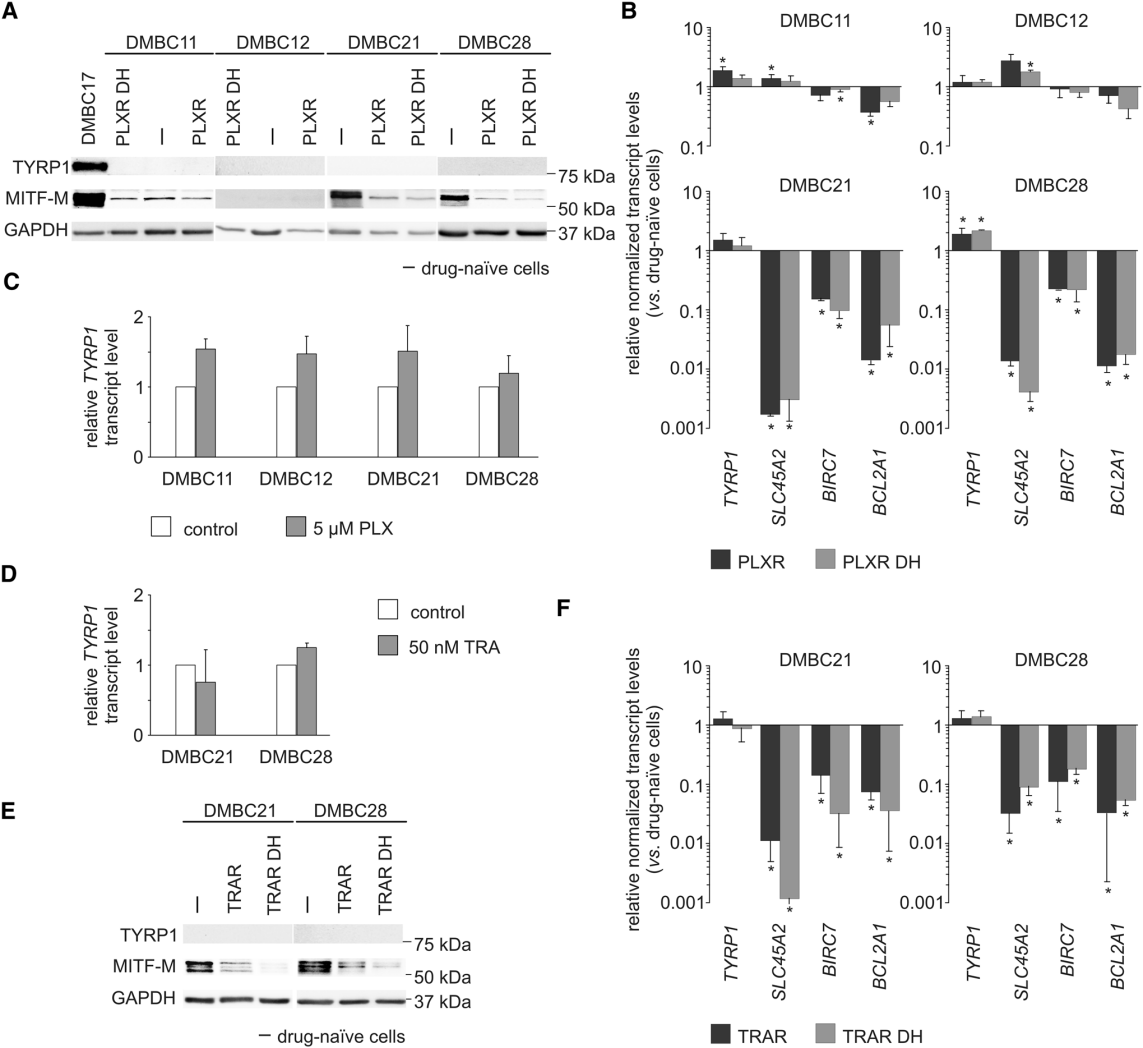
*TYRP1* expression is unaltered at the mRNA level and remains undetectable at the protein level during development of resistance to vemurafenib (PLX) and trametinib (TRA) in BRAF^V600E^ melanoma cells regardless of changes in the MITF-M level. **a** Representative Western blot images showing comparison of TYRP1 and MITF-M protein levels in drug-naïve (–) vs. vemurafenib-resistant (PLXR) cell lines. The levels of TYRP1 and MITF-M in vemurafenib-resistant melanoma cells subjected to drug discontinuation (‘drug holiday’; PLXR DH) are included. GAPDH was used as a loading control. DMBC17 cell lysate was used in parallel as a positive control for TYRP1 and MITF-M staining. **b** Comparison of expression of *TYRP1*, *SLC45A2*, *BIRC7* and *BCL2A1* in vemurafenib-resistant BRAF^V600E^ melanoma cell lines either exposed to drug or after drug discontinuation (‘drug holiday’), relative to expression in their drug-naïve counterparts. Bars represent mean values ± SD, *n* = 3, *p ≤ 0.05. **c** Expression of *TYRP1* at the mRNA level in drug-naïve melanoma cells exposed to vemurafenib for 22 h, relative to its expression in untreated cells. Bars represent mean values ± SD, *n* = 3. **d** Expression of *TYRP1* at the mRNA level in drug-naïve melanoma cells exposed to trametinib for 22 h, relative to its expression in control cells. Bars represent mean values ± SD, *n* = 3. **e** Representative Western blot images showing comparison of TYRP1 and MITF-M protein levels in drug-naïve (–) vs. trametinib-resistant (TRAR) cell lines, also subjected to drug discontinuation (‘drug holiday’; TRAR DH) are included. GAPDH was used as a loading control. **f** Comparison of expression of *TYRP1*, *SLC45A2*, *BIRC7* and *BCL2A1* in TRAR cell lines either exposed to drug or after drug discontinuation (TRAR DH), relative to expression in their drug-naïve counterparts. Bars represent mean values ± SD, *n* = 3. Expression of indicated genes in **b**–**d** and **f** was assessed by qRT-PCR and normalized to the expression of a reference gene *RPS17*

First, we modeled the vemurafenib resistance in four BRAF^V600E^ melanoma cell lines. During development of resistance, the MITF-M protein level was substantially diminished in MITF-M^high^ cells and remained low/undetectable in MITF-M^low^ melanoma cells (Fig. 2a). TYRP1 protein could not be detected in vemurafenib-resistant cells, similarly as in their drug-naïve counterparts (Fig. 2a). Changes in MITF-M level were reflected in its activity shown as downregulation of three MITF-M-dependent genes, *SLC45A2*, *BIRC7* and *BCL2A1* (Fig. 2b). On the contrary, TYRP1 transcript level was stable in vemurafenib-resistant cell lines regardless of changes in the MITF-M level (Fig. 2b). This further supports the notion that *TYRP1* expression is MITF-M-independent. Interestingly, when vemurafenib-resistant cells were subjected to drug removal (“drug holiday”) for 10 days, MITF-M level and expression of all four tested genes remained unaffected (Fig. 2a, b). TYRP1 transcript levels, which were almost the same in drug-naïve, vemurafenib-resistant and on-drug-holiday BRAF^V600E^ melanoma cells, were not markedly altered also in cells only shortly exposed to vemurafenib (Fig. 2c). Similarly, short treatment with trametinib, a MEK1/2 inhibitor, did not affect substantially the transcript level of TYRP1 in MITF-M^high^ cell lines, DMBC21 and DMBC28 (Fig. 2d). Reduction of *MITF* expression observed after development of resistance to trametinib in those cell lines was even potentiated after drug cessation (Fig. 2e). While this was associated with a significant downregulation of *SLC45A2*, *BIRC7* and *BCL2A1* (Fig. 2f), TYRP1 transcript level was not significantly altered (Fig. 2f).

### TYRP1 expression remains stable in the majority of melanomas during development of resistance to BRAF^V600^ and MEK1/2 inhibitors

**Fig. 3:**
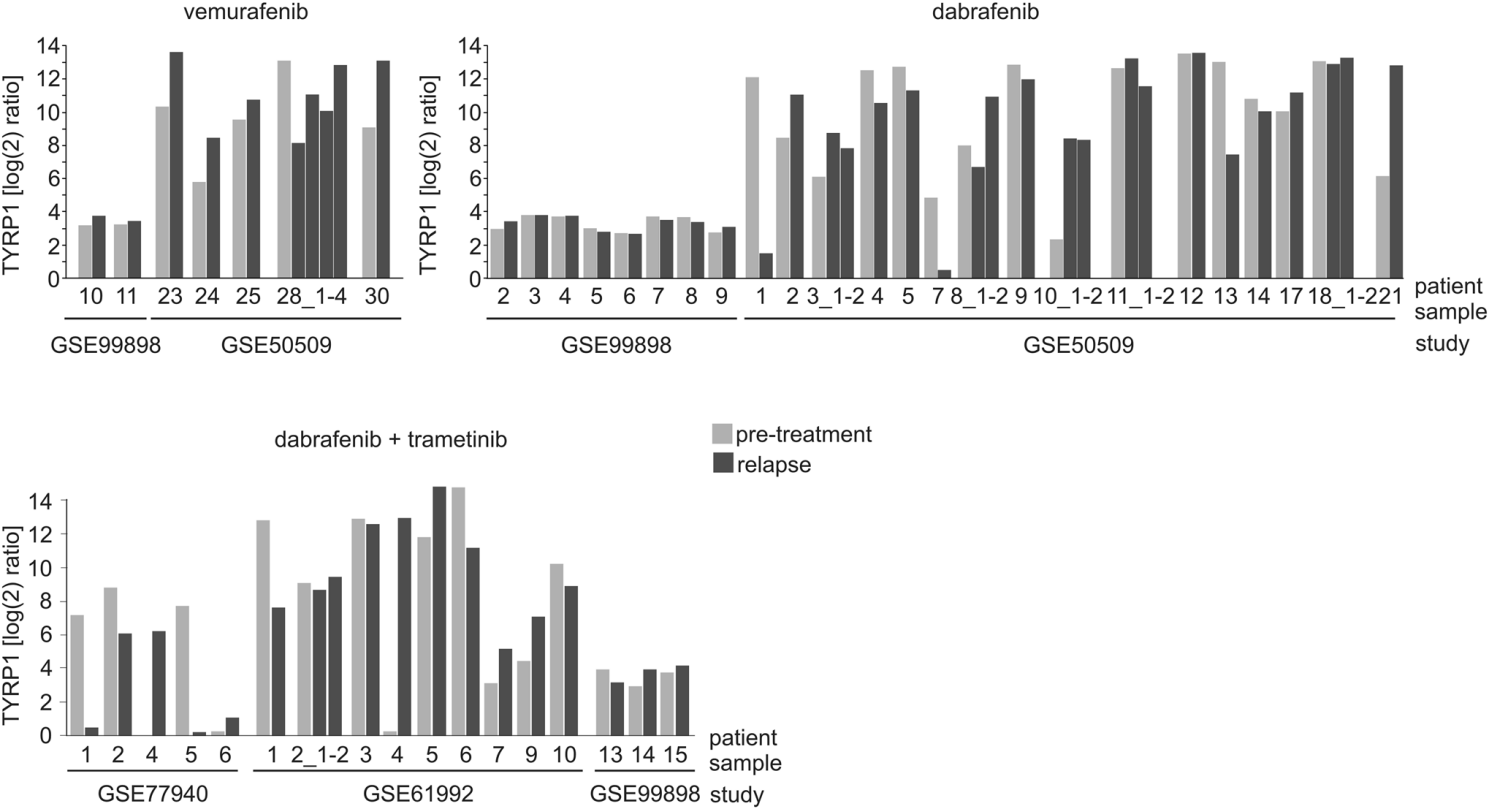
*TYRP1* expression at the mRNA level is not markedly changed in the majority of the relapsed patients who developed resistance to BRAF^V600^ inhibitors, vemurafenib or dabrafenib, or combined BRAF^V600^ and MEK1/2 inhibitors, dabrafenib and trametinib, in comparison to its expression before treatment. Data showing TYRP1 mRNA levels in paired melanoma specimens, pretreatment and post-relapse, are presented as light gray columns and dark gray columns, respectively. For some patients, more than one relapsed samples were examined. Results are expressed as log_2_ ratios normalized to the mean intensity of pretreatment specimens. Data were obtained from NCBI GEO (https://www.ncbi.nlm.nih.gov/geo/), and study identifiers are indicated.

TYRP1 mRNA levels in melanomas from patients who developed resistance to targeted therapeutics were examined using publicly available data sets. The majority of relapsed melanomas did not show any evidence of TYRP1 transcript loss (Fig. 3). Among seven patients who developed resistance to vemurafenib only one exerted reduced *TYRP1* expression in three out of four relapsed specimens. Among 24 patients treated with dabrafenib, another BRAF^V600^ inhibitor, the development of resistance could be linked with reduced *TYRP1* expression only in 3 patients that relapsed, when compared with the pretreatment specimens from the same patients. In other relapsed specimens it was either markedly increased (2 specimens) or remained unchanged (19 specimens). Similar trend was observed when TYRP1 transcript level was analyzed in samples from patients treated with a combination of dabrafenib and trametinib. Among 17 patients, diminution of *TYRP1* expression was found only in 4 patient specimens, while TYRP1 mRNA level increased in 6 samples and remained unaltered in other 7 specimens (Fig. 3). This analysis further supports the notion that the TYRP1 transcript level is stable also in clinical samples of relapsed melanomas that are resistant to BRAF^V600^ and BRAF^V600^ + MEK1/2 inhibitors.

## Discussion

Elevated TYRP1 mRNA levels were detected in metastatic melanoma biopsies [3, 16], and correlated with poor overall patient survival [10–12, 28]. Unlike TYRP1 mRNA, TYRP1 protein did not correlate with overall survival, and TYRP1 protein was not detected in half of the melanoma samples expressing the TYRP1 transcript [11, 12, 28].

Our results indicate that TYRP1 protein is not detectable in the majority of samples that express *TYRP1* at the transcript level. Moreover, mutation leading to BRAF^V600E^ might be connected with a medium level of TYRP1 mRNA and undetectable TYRP1 protein, whereas mutation leading to either NRAS^Q61R^ or HRAS^Q61R^ might be associated with high levels of TYRP1 transcript and protein. It would be interesting to check whether there is any correlation between the molecular type of melanoma (m*BRAF*, m*RAS*, m*NF1*, or triple wild type) [38] and *TYRP1* expression.

The high level of TYRP1 mRNA promotes proliferation and tumor growth irrespective of the protein level [17]. In our study, there was no strong association between TYRP1 mRNA level and cell proliferation rate. Melanoma cell lines with the highest proliferation rate were among lines expressing TYRP1 transcript at a medium level, whereas those with the highest expression of *TYRP1* at the transcript and protein levels exerted one of the lowest proliferation rates. However, our results are in agreement with the findings of Gilot et al. [17] when cells lacking detectable TYRP1 protein and expressing TYRP1 mRNA at the level below the median value are exclusively considered. This suggests that the proliferation rate may be connected with TYRP1 mRNA level within this narrow range of *TYRP1* expression.

The level of TYRP1 mRNA is a consequence of the transcriptional and post-transcriptional regulation [13–15, 18, 31, 41, 43]. It is possible that regulation of *TYRP1* expression in MITF-M^low^ melanoma cells is stabilized by other transcription factors or regulatory mechanisms. The single-nucleotide polymorphism rs683 located in the 3′-untranslated region of *TYRP1* was connected with reduced binding of miR-155 and its mRNA decay activity [10, 31]. miRNA-155 expression is down-regulated in the majority of melanoma cell lines in comparison to melanocytes [30]. Therefore, influence of miR-155 on the TYRP1 transcript stability might be diminished during melanoma development. It has also been shown that miR-16, which interplays with miR-155, participates in the stabilization of TYRP1 mRNA [36].

*TYRP1* expression is MITF-M-dependent in melanocytes [14]. A significant correlation between TYRP1 mRNA level and MITF level was found also in a subset of melanoma cell lines [11, 41]. Our results are contradictory to these findings as: (i) *TYRP1* expression was similar in MITF-M^low^ and MITF-M^high^ melanoma cells and (ii) MITF-M loss accompanying the development of drug resistance in MITF-M^high^ cells, did not influence *TYRP1* expression. Interestingly, our results are consonant with an earlier study showing lack of significant changes in *TYRP1* expression upon Mitf-transfection of Mitf^low^ SK-MEL-28 melanoma cells [27]. The expression of other genes, *BCL2A1*, *SLC45A2* and *BIRC7*, was markedly upregulated upon Mitf-transfection in that study [27], indicating that only the *TYRP1* promoter was non-responsive to functional exogenous MITF.

The role of RNAs as endogenous miR sponges has been demonstrated [2, 7, 19, 25, 29, 39]. TYRP1 mRNA on its non-canonical miRNA response elements can sequester miR-16 and de-repress targets of miR-16 [17]. For the first time, we have shown that the TYRP1 transcript level is not affected during both acute response and development of resistance to vemurafenib and trametinib, which suggests that the role of TYRP1 mRNA as endogenous ‘miR sponge’ is preserved in resistant cells. Our findings are supported by the analysis of publicly available data on *TYRP1* expression in melanoma samples from patients before treatment and at the time of tumor progression due to development of resistance to targeted therapeutics. This analysis confirms stable expression of *TYRP1* in melanoma cells irrespective of their sensitivity to BRAF^V600^ and MEK1/2 inhibitors.

## Conclusion

A ‘sponge’ activity of TYRP1 transcript in melanoma cells resistant to BRAF^V600E^ and MEK1/2 inhibitors was not considered until now. Our results proving that *TYRP1* expression is MITF-M-independent in melanoma cells suggest that regardless of the mechanisms that are responsible for the stable level of TYRP1 mRNA in melanoma cells, therapies developed to terminate a sponge activity of TYRP1 transcript might be extended to patients that relapse with vemurafenib/trametinib-resistant disease.
